# Defective Tool Embodiment in Body Representation of Individuals Affected by Parkinson’s Disease: A Preliminary Study

**DOI:** 10.3389/fpsyg.2018.02489

**Published:** 2019-01-07

**Authors:** Federica Scarpina, Nicola Cau, Veronica Cimolin, Manuela Galli, Lorenzo Priano, Alessandro Mauro

**Affiliations:** ^1^Istituto Auxologico Italiano, IRCCS, Divisione di Neurologia e Neuroriabilitazione, Ospedale San Giuseppe, Piancavallo (VCO), Italy; ^2^Department of Electronics, Information and Bioengineering, Politecnico di Milano, Milan, Italy; ^3^IRCCS San Raffaele Pisana, Tosinvest Sanità Roma, Rome, Italy; ^4^Department of Neuroscience “Rita Levi Montalcini”, University of Turin, Turin, Italy

**Keywords:** Parkinson’s disease, tool embodiment, body representation, action, multisensory integration, body schema

## Abstract

When efficiently used for action, tools become part of the body, with effect on the spatial-temporal movement parameters and body size perception. Until now, no previous investigation has been reported about tool embodiment in Parkinson’s disease (PD), which is a neurological disease characterized by several sensory and motor symptoms affecting body and action. We enrolled 14 individuals affected by PD and 18 healthy individuals as controls. We studied the spatial-temporal parameters on self-paced free pointing movement task, *via* an optoelectronic system, before and after a short training in which a 27-cm long rod was used to point toward a far target. Moreover, we investigated changes in estimation of arm length through the Tactile Estimation Task. After the tool-use training, controls showed changes in spatial-temporal parameters: they were slower to perform movements and reported a higher value of deceleration than the baseline. However, such a difference did not emerge in the PD individuals. In the Tactile Discrimination Task, no difference emerged before and after the tool-use training in both groups. Our results were suggestive of possible difficulties of the tool embodiment process in PD. We discussed our results in relation to aberrant multisensory integration as well as in terms of the effect of PD sensory and motor symptoms on body schema plasticity. The present study points at a novel way to conceive PD sensory motor signs and symptoms in terms of their effect on individuals’ body representation.

## Introduction

One of the most peculiar characteristics of a human being is the capability to use tools for acting in the environment. For example, we can use a rod to indicate something that is out of our reaching space: the tool makes near what would otherwise be unreachable. When efficiently used, tools become part of our body; in other words, it is *embodied* (Maravita and Iriki, 2004; Longo and Serino, 2012; Miller et al., 2018) with effects on action, perceptions, and cognitive capacities (Cardinali et al., 2009). For instance, after a tool-use training, healthy individuals perceive their arm as longer than before; moreover, changes in spatial-temporal parameters of motor behavior are observed (Cardinali et al., 2009). These “changes are compatible with the notion of the inclusion of tools in the ‘Body Schema,’ as if our own effector (e.g. the hand) were elongated to the tip of the tool” (Maravita and Iriki, 2004), where the term *body schema* (Gallagher, 2005) refers to the dynamic sensory-motor body representation derived from the integration of multiple sensory bodily inputs and is used to plan and execute actions (Gallagher, 2005; Dijkerman and De Haan, 2007). Body schema is known to be a plastic representation (Gallagher, 2005; Giummarra et al., 2008); not only it is constantly updated in relation to the online incoming sensory input, but also it changes in order to embody significant objects (Dijkerman and De Haan, 2007; Giummarra et al., 2008). Then, the adoption of an experimental paradigm grounded on tool embodiment has allowed to investigate the plasticity of body schema (Martel et al., 2016) in healthy individuals (Cardinali et al., 2009; Canzoneri et al., 2013) and in pathological conditions (Giummarra et al., 2008), such as amputees who use a prosthesis (Mayer et al., 2008), individuals with spinal cord injury who use the wheelchair (Pazzaglia et al., 2013), and brain-damaged patients (Garbarini et al., 2015) (for details, see Giummarra et al., 2008).

In the present work, we aimed to provide a first and preliminary investigation about tool embodiment in Parkinson’s disease (PD). It is a neurological syndrome characterized by several motor and sensory symptoms, such as akinesia and bradykinesia, tremor and rigidity, and postural instability (Bereczki, 2010). These symptoms are due to the dysfunction of neural structures responsible for movement selection, coordination, and execution (see Moustafa et al., 2016 for a review); then, in PD, body and action are primarily affected. Interestingly, in the literature, preliminary but not conclusive evidence has been reported about changes in sensory bodily function ranging from primary sensory perception to the complex integration of multiple sensory and motor inputs in PD (Abbruzzese and Berardelli, 2003; Avanzino et al., 2018); however, the effect of PD symptoms on the bodily self (i.e. body awareness, sense of agency, and proprioception) (Blanke et al., 2015) and body representation is still in infancy.

In order to verify if a tool can be efficiently embodied in body representation in PD, we studied motor parameters of self-paced free pointing movements, before and after a short training in which a rod was used as a tool to point toward a far target. Moreover, we verified if the tool embodiment changed the cognitive representation of the arm used to handle the tool through the Tactile Estimation Task (Scarpina et al., 2014): in this task, participants estimated the distance between two tactile stimuli presented simultaneously on the arm. This judgment allows to infer the internal body representation of physical size of the arm (Serino and Haggard, 2010), namely how long do the participants estimate their arm. If the tool is correctly embodied, the arm should be represented as longer of its physical dimension, and consequently, the distance between the two tactile inputs might be perceived larger than the real gap.

Considering the previous studies (Maravita and Iriki, 2004; Cardinali et al., 2009, 2011; Longo and Serino, 2012), if PD affects the tool embodiment process, we might expect no changes in affected individuals’ motor parameters of pointing movements, while in the healthy individuals, such a change should be emerged. Similarly, in the Tactile Estimation Task, no difference might be found before and immediately after the tool-use training in affected individuals’ performance, whereas the healthy individuals might judge their arm longer after the tool-use training than the baseline condition, as an effect of a correct embodiment of the tool in their body representation. Nevertheless, possible dissociations might be emerged between the two tasks, since they rely on different components (one devoted to action and the other to perceptual description) of body representation (Gallagher, 2005; Dijkerman and De Haan, 2007).

## Materials and Methods

The study was approved by the ethical committee of the IRCCS Istituto Auxologico Italiano, and it was performed in compliance with Declaration of Helsinki’s (World Medical Association, 1991) ethical principles. All participants were volunteers who gave informed written consent, were free to withdraw at will, and were naïve to the rationale of the experiment.

### Participants

Fourteen individuals affected by PD (seven patients showing to have the right body side most predominantly affected by PD; seven patients, the left body side; *age* in years *M* = 66; *standard deviation* = 8; *education* in years *M* = 9; *SD* = 3) were recruited at the Division of Neurology and Neurorehabilitation, IRCCS Istituto Auxologico Italiano, San Giuseppe Hospital in Piancavallo (VCO, Italy).

All participants were right handers. They had been diagnosed as having PD (mean years from diagnosis *M* = 7, *SD* = 3) according to the Hoehn and Yahr’s (1967) classification. The PD group reported a mean score of 30 (*SD* = 13) on the unified Parkinson’s disease rating scale (UPDRS) (Fahn and Elton, 1987). Exclusion criteria were the evidence of other neurological (e.g., ictus, traumatic brain injury; dementia) or pathological conditions (e.g., psychiatric syndromes; POTS). Moreover, a threshold of 24 (Lezak et al., 2004) for Mini Mental State Examination (MMSE) (Folstein et al., 1975) was adopted as an inclusion criterion. Details are reported in Table 1.

**Table 1:** Demographical and clinical details of individuals affected by PD.

ID	Sex	Age	Education	Duration of disease	UPDRS score (on)	H and Y stage	Most-affected side
1	F	72	5	6	37	3	L
2	M	76	5	5	28	2	R
3	M	56	8	5	28	2	L
4	F	72	13	6	43	3	L
5	F	75	8	6	12	1	L
6	M	63	5	9	57	4	L
7	F	74	11	11	22	2	R
8	M	68	8	7	41	2	R
9	F	56	13	8	31	2	R
10	F	77	13	4	21	2	L
11	M	54	13	6	19	2	L
12	F	67	5	17	27	2	L
13	F	57	13	9	13	2	R
14	F	69	13	8	46	3	R
**Mean**		66	9	7	30		
(*SD*)		(8)	(3)	(3)	(13)		

M = male and F = female. Age, education and duration of disease expressed in years. L = left body side. R = right body side. Means and standard deviations (*SD*) are reported in the lower part of the table.

All individuals with PD were tested when they were in a self-reported ‘*on*’ state of medication, meaning when symptoms were efficiently managed by drugs, even though with negative effects on movement control (Cenci, 2007) and proprioception (O’Suilleabhain et al., 2001). In fact, when individuals are in an *‘off’* state, symptoms such as tremor, rigidity, and slowness, as well as difficulty in attention, feeling to be completely blocked, anxiety, and pain emerge or worsen (Ahlskog and Muenter, 2001; Fahn et al., 2004), limiting not only the interpretation of the results, but also the patient’s compliance to perform the task.

Eighteen healthy right-handed participants (*age* in years *M* = 48; *SD* = 14; *education* in years *M* = 15; *SD* = 3) without sensory, neurological, or psychiatric impairments were recruited through personal contact with the researchers or word-of-mouth.

Individuals with PD were significantly older than healthy controls [*age U*(32) = 212; *p* < 0.001], and they had significantly fewer years of *education* [*U*(32) = 37.5; *p* < 0.001].

### Experimental Task

**Figure 1:**
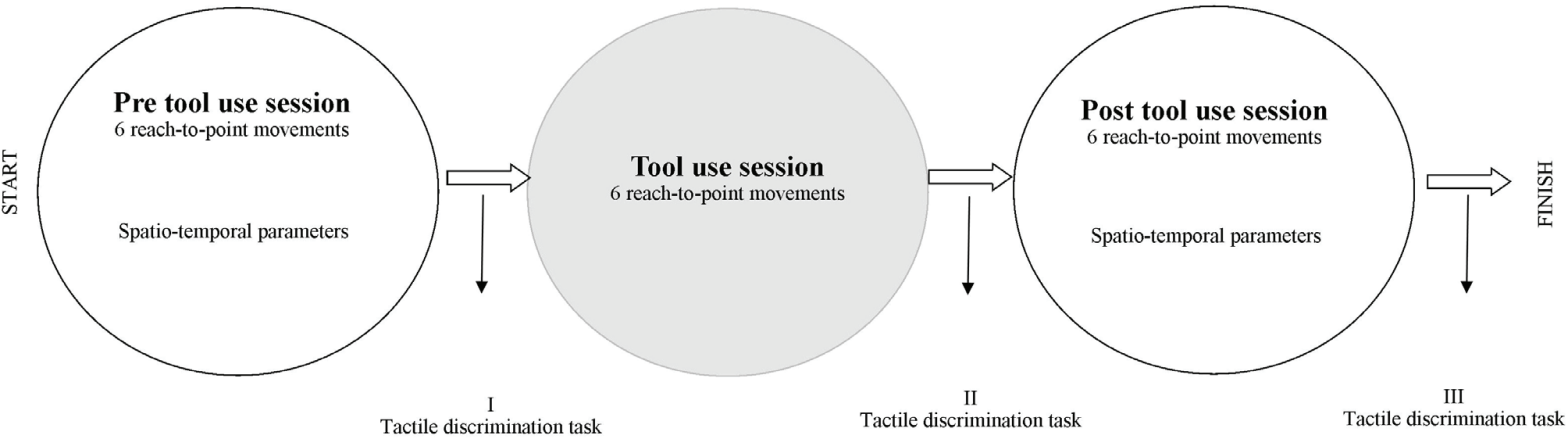
Graphical representation of the timeline of the experiment.

In Figure 1, a timeline of the experiment is shown.

#### Pointing Movement Tasks

Participants were comfortably seated at the table, with their body midline aligned with the central midline of the table. The experiment had three phases: a *pre*- (i.e., the baseline) and *post-tool-use session* spaced out by the tool-use session (Figure 1). In the *pre-* and *post*-tool-use sessions, participants performed six reach-to-point movements. The target was a black dot placed at a distance equal to 80% of the arm length from the body. Thus, for each participant, the arm length was recorded. Participants were required to extend their arms in the straight-ahead direction, at shoulder height; the horizontal distance between the acromion and the middle finger was measured. During the pointing movement task, the other hand was placed in the rest position in line with the corresponding starting point. The time of the movements was self-paced. The experimenter visually checked that participants completed the six movements.

In the *tool-use* session, participants were asked to perform six movements using a stick, 27 cm long and weighing 4 g, in order to reach the visual target with the same arm used in the *pre-* and *post*-tool-use session. In this condition, the dot was placed at a distance equal to 27 cm (i.e. equal length of the stick) from the target used in the *pre-* and *post*-tool-use sessions, far away with respect to the body midline; thus, the target was placed outside the arm-reaching distance. Participants were instructed to reach the target and to touch it, before going back to the starting point (i.e., the rest position in which individual’s forearm made a 90° angle with the arm and the shoulder). During the *tool-use* session, the other hand was placed in the rest position in line with the corresponding starting point. The time of the movements was self-paced. The experimenter visually checked that participants completed the six movements.

The 3D-movement acquisition was conducted using an optoelectronic system with passive markers (VICON, Oxford Metrics Ltd., Oxford, UK) for kinematic movement evaluation. The optoelectronic system performed a real-time processing of images from six fixed infrared cameras (a sampling rate of 100 Hz) to extract the reflectance of a passive marker (with a diameter of 15 mm) that was positioned on the participants’ index fingers (Cimolin et al., 2007).

#### Tactile Estimation Task

After all the three sessions (*pre*-tool use, *tool-use* training, and *post-*tool use) of the Pointing Movement Task, a modified version of the Tactile Estimation Task (Scarpina et al., 2014) was performed. Participants were with eyes closed for the duration of the task. The experimenter lightly pressed the two pointers of a caliper on the participants’ ventral side of forearm, following the longitudinal axis. Participants were asked to estimate the distance between the two tactile stimuli by varying the separation between the thumb and the index hand fingers of the non-stimulated arm. The distance between the two pointers was set at 7 cm in all repetitions. The tactile stimulation was repeated seven times for the session: overall 21 trials, about which 7 immediately after the *pre*-tool-use session (Figure 1 – I), 7 immediately after the *tool-use* training (Figure 1 – II), and finally 7 after the *post*-tool-use session (Figure 1 – III), were performed. In line with previous studies (Cardinali et al., 2009, 2011), the embodiment of tools might emerge as a larger error in the second measurement, i.e., immediately after the *tool-use* training, with respect to the baseline, meaning the first measurement done after the *pre*-tool-use session. In other words, after the *tool-use* training, participants might evaluate their arms as longer as the baseline. The third measurement, i.e., after the second pointing movement session (the *post*-tool-use session), gave us the opportunity to verify if possible changes in tactile estimation observed in Session II (after *tool-use* training) can still be observed also when the movements were performed without any tool or, on the other hand, if the last action restores the original bodily estimation.

The entire experimental task was performed twice, with both right and left hands. The order of hands was counterbalanced between participants.

### Analyses

A *post hoc* power analysis was conducted using the software package GPower 3.0.1. A sample size of 28 was used (14 participants for two groups); moreover, the alpha level used for this analysis was *p* < 0.05. The *post hoc* analyses revealed that the statistical power for this study was 0.99 for detecting a medium effect size (*d* = 0.5), whereas it was 0.1 for a large effect size (*d* = 0.8).

#### Pointing Movement Task

**Figure 2:**
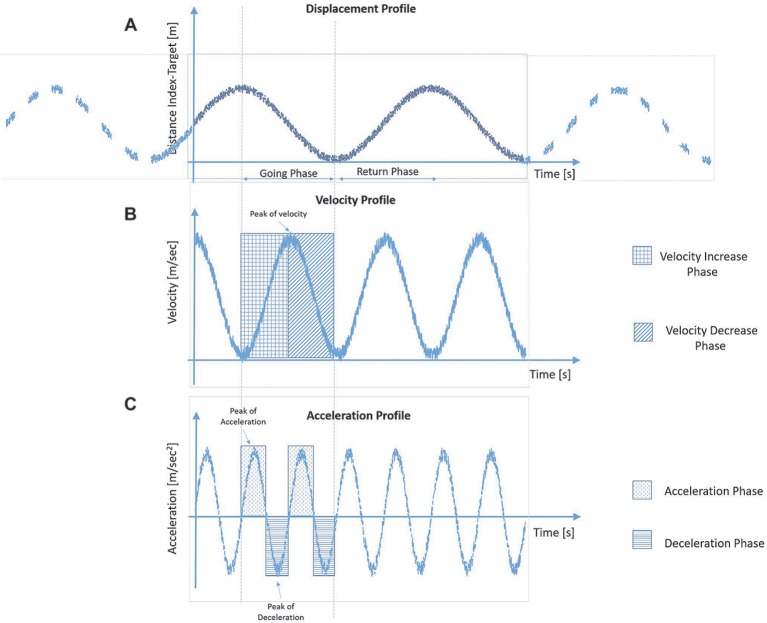
Graphical representation of the typical profiles of displacement **(A)**, velocity **(B)** and acceleration **(C)** of the finger marker during the going phase in the reach-to-pointing task.

For each trial, spatio-temporal parameters relative to the pointing movements were measured in the *pre*- (i.e. the baseline) and *post-*tool-use conditions. Each parameter was referred to the going phase, and it was calculated using the 3D coordinate of the index finger marker. During the going phase, the distance between the marker of the finger and the target decreases (Figure 2A), and its value is close to zero once the participant reached the target. When the velocity profile is taken into account, it increases its value until a peak of velocity—maximum value. Then, the velocity value reduces quickly to guarantee the proper accuracy during the adjustment phase (Figure 2B). Velocity and acceleration profiles are strictly related: the latter is the derivative of velocity with time, and velocity itself is the derivative of displacement with time. Acceleration achieves its maximum during the increase phase of the velocity and gets zero in correspondence with the peak of velocity. Then, the velocity profile decreases, and the acceleration changes its sign—negative value—and we observe a deceleration phase (Figure 2C).

Thus, the following parameters are defined as follows: *movement time* from the starting point to the target, expressed in s; *mean velocity,* defined as the average velocity of the finger marker during the going phase; and *peak of velocity*, defined as the maximum velocity of the finger marker during the going phase, in m/s; *mean acceleration and peak of acceleration* in m/s^2^; *mean deceleration and peak of deceleration* in m/s^2^. The data relative to the six trials for each condition and hands were collapsed together, since preliminary analyses revealed no difference between right and left arms for healthy controls as well as no difference between affected or non-affected arm for PD patients when the lateralization of symptoms was taken into account. A repeated measure analysis of variance with the within-subject factor of *Time* (pre-tool use vs post-tool use) and the between-subject factor of *Group* (PD group vs control group) was performed for each motor parameter. If the interaction was significant, Bonferroni-corrected estimated marginal mean comparisons were applied.

#### Tactile Estimation Task

The difference between the estimated distance and the physical distance between the two pointers of the caliper (7 cm) was computed for each trial, representing the *error*. A negative error indicated an underestimation of the perceived distance; a positive error indicated an overestimation of the perceived distance. A repeated measure ANOVA with the within-subject factor of *Time* (pre-tool use, tool-use training, and post-tool use) and the between-subject factor of *Group* (PD group vs control group) was performed, applying Bonferroni-corrected estimated marginal mean comparisons in the case of significant interaction.

#### The Role of Age

Considering that the two groups were significantly different in terms of *age* with possible effects on embodiment (Costello et al., 2015; Costello and Bloesch, 2017), for both tasks (Movement Pointing Task and Tactile Estimation Task), the analysis was run again introducing the factor *Age* as a covariate for those parameters about which a significant main effect of *Group* of interaction with *Time* was found in the previous analyses.

#### The Role of Clinical Characteristics

Only for the group of individuals affected by PD, the possible relationship between the clinical characteristics of *Duration of Disease* and *UPDRS motor score* and the spatio-temporal parameters relative to the pointing movements measured in the pre- and post-tool-use conditions was explored through Spearman’s rank correlation coefficient. Moreover, the possible difference in all spatio-temporal parameters between PD individuals with a left lateralization of symptoms and those with a right lateralization was explored through the Mann–Whitney *U* test. The same analyses were conducted about the three experimental sessions (*pre*-tool use, *tool-use* training, and *post*-tool use) of the Tactile Estimation Task.

## Results

All participants completed the task as well as the tool-use training.

### Pointing Movement Task

**Figure 3:**
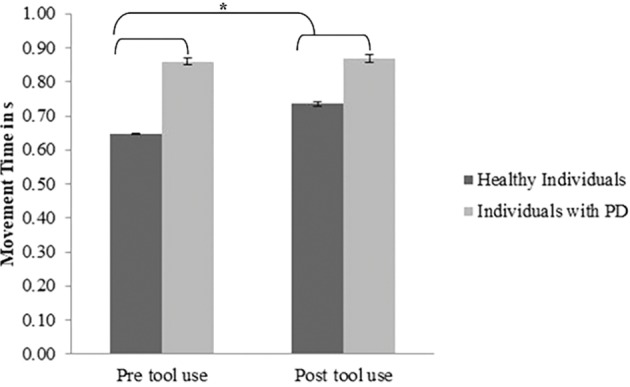
*Movement Time* expressed in s, mean values, and standard error (vertical line) in pre-tool-use and post-tool-use conditions by group (dark grey = healthy individuals; light grey = individuals with PD) are shown. Asterisk denotes *p* < 0.05 in the *post hoc* comparisons.

*Movement time*: A significant main effect of *Group* (PD group *M* = 0.859; *SD* = 0.03; control group *M* = 0.69; *SD* = 0.02) emerged [*F*(1, 30) = 20.82; *p* < 0.001; partial *η* ^2^ = 0.99]: PD patients required significantly more time to perform movements than the controls. Moreover, a main effect of *Time* (pre-tool use *M* = 0.739; *SD* = 0.142; post-tool use *M* = 0.794; *SD* = 0.093) emerged [*F*(1, 30) = 7.3; *p* = 0.011; partial *η* ^2^ = 0.196]: in the post-tool-use condition, individuals required more time to perform movements than the baseline. Interestingly, the *Group × Time* interaction was significant [*F*(1, 30) = 4.72; *p* = 0.038; partial *η* ^2^ = 0.55]; while the healthy individuals required significantly more time in the post-tool-use condition than the baseline (*p* = 0.001), this difference did not emerge in PD patients’ performance (*p* = 0.72). Moreover a significant difference emerged between the two groups in the pre-tool use (*p* < 0.001) and post-tool use (*p* = 0.011) (Figure 3); in both conditions, PD patients required more time to perform the movements.

*Mean velocity*: A main effect of *Group* [*F*(1, 30) = 9.55; *p* = 0.004; partial *η* ^2^ = 0.84] emerged: PD patients (*M* = 0.399; *SD* = 0.09) were significantly slower than the healthy participants (*M* = 0.475; *SD* = 0.07). No main effect of *Time* (pre-tool use *M* = 0.442; *SD* = 0.09; post-tool use *M* = 0.46; *SD* = 0.1) [*F*(1, 30) = 2.85; *p* = 0.1; partial *η* ^2^ = 0.087] or a significant *Group × Time* interaction [*F*(1, 30) = 3.52; *p* = 0.07; partial *η* ^2^ = 0.1] emerged.

*Peak of velocity*: A main effect of *Group* [*F*(1, 30) = 12.64; *p* = 0.001; *η* ^2^ = 0.93] emerged: PD patients (*M* = 0.68; *SD* = 0.03) reported a significant lower peak of velocity than the control group (*M* = 0.89; *SD* = 0.02). Moreover, a main effect of *Time* [*F*(1, 30) = 16.71; *p* < 0.001; partial *η* ^2^ = 0.35] emerged: in post-tool-use condition (*M* = 0.838; *SD* = 0.165), a significantly higher peak of velocity was observed than the baseline (*M* = 0.79; *SD* = 0.16). The *Group × Time* interaction was not significant [*F*(1, 30) = 0.36; *p* = 0.55; partial *η* ^2^ = 0.12].

*Mean acceleration*: A main effect of *Group* [*F*(1, 30) = 22.89; *p* < 0.001; partial *η* ^2^ = 0.43] was found: PD patients (*M* = 1.997; *SD* = 0.09) reported a significant lower acceleration than the control group (*M* = 2.719; *SD* = 0.12). Moreover, a main effect of *Time* emerged [*F*(1, 30) = 6.72; *p* = 0.015; partial *η* ^2^ = 0.18], since in the post-tool-use condition (*M* = 2.48; *SD* = 0.62), the acceleration was higher than the baseline (*M* = 2.32: *SD* = 0.52). No significant *Group × Time* interaction [*F*(1, 30) = 0.32; *p* = 0.85; partial *η* ^2^ = 0.01] emerged.

*Peak of acceleration*: A main effect of *Group* emerged [*F*(1, 30) = 44.87; *p* < 0.001; *η* ^2^ = 0.59]: PD patients (*M* = 5.45; *SD* = 0.22) reported a significant lower peak of acceleration than the control group (*M* = 8.271; *SD* = 0.35). No significant main effect of *Time* (pre-tool use *M* = 6.99; *SD* = 1.99; post-tool use *M* = 7.08; *SD* = 1.81) [*F*(1, 30) = 0.47; *p* = 0.49; *η* ^2^ = 0.59], (*p* = 0.015) or significant *Group × Time* interaction [*F*(1, 30) = 3.123; *p* = 0.087; partial *η* ^2^ = 0.094] emerged.

**Figure 4:**
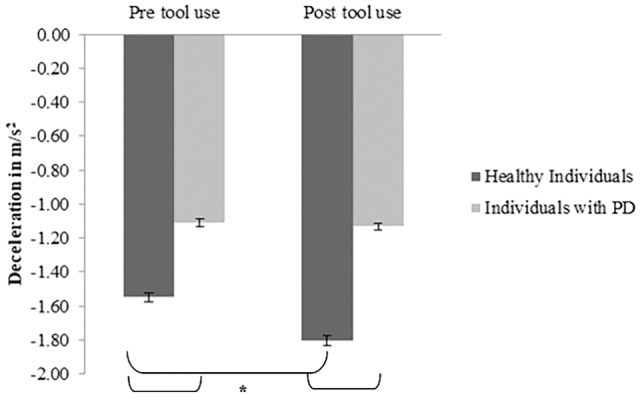
*Deceleration* expressed as m/s^2^, mean values, and standard error (vertical line) in pre-tool-use and post-tool-use conditions by group (dark grey = healthy individuals; light grey = individuals with PD) are shown. Asterisk denotes *p* < 0.05 in the *post hoc* comparisons.

*Mean deceleration*: A main effect of *Group* emerged [*F*(1, 30) = 13.97; *p* = 0.01; partial *η* ^2^ = 0.31] (PD group *M* = −1.11; *SD* = 0.08; control group *M* = −1.675; *SD* = 0.11). A main effect of *Time* [*F*(1, 30) = 14.21; *p* = 0.001; partial *η* ^2^ = 0.32] was found: indeed in the post-tool-use condition (*M* = −1.5; *SD* = 0.56), the deceleration was higher than the baseline. Interestingly, the *Group × Time* interaction was significant [*F*(1, 30) = 10.2; *p* = 0.003; partial *η* ^2^ = 0.25]; while the healthy individuals reported a significantly higher value of deceleration in the post-tool-use condition than the pre-tool-use condition (*p* = 0.001), this difference did not emerge in individuals with PD patients’ performance (*p* = 0.703). Moreover, a significant difference emerged between the two groups in the pre-tool use (*p* < 0.005) and post-tool use (*p* < 0.001) conditions; in both experimental sessions, PD patients showed lower deceleration than controls (Figure 4).

*Peak of deceleration*: A main effect of *Group* [*F*(1, 30) = 24.73; *p* < 0.001; partial *η* ^2^ = 0.04] emerged: PD patients (*M* = −3.24; *SD* = 0.1) reported a significant lower peak of deceleration than the control group (*M* = −4.456; *SD* = 0.21). Also, a main effect of *Time* [*F*(1, 30) = 9.87; *p* = 0.004; partial *η* ^2^ = 0.24] emerged: in post-tool-use condition (*M* = −4.05; *SD* = 0.96), a higher peak of deceleration was found than the baseline (*M* = −3.79; *SD* = 0.91). The *Group × Time* interaction [*F*(1, 30) = 0.49; *p* = 0.48; partial *η* ^2^ = 0.016] was not significant.

### The Role of Age on the Performance in the Pointing Movement Task

We run again the analyses, controlling the effect of *Age.*

*Movement time*: The main effect of *Group* still remained significant [*F*(1, 29) = 13.73; *p* = 0.001; partial *η* ^2^ = 0.32): PD patients (adjusted *M* = 0.868; *SD* = 0.03) were significantly slower than the healthy participants (adjusted *M* = 0.688; *SD* = 0.02). Instead, neither main effect of *Time F*(1, 29) = 0.78; *p* = 0.38; partial *η* ^2^ = 0.026) nor the *Group × Time* interaction [*F*(1, 29) = 2.23; *p* = 0.1; partial *η* ^2^ = 0.072] was significant.

*Mean velocity*: The main effect of *Group* still remained significant [*F*(1, 29) = 4.37; *p* = 0.045; partial *η* ^2^ = 0.13], since PD patients (adjusted *M* = 0.406; *SD* = 0.026) were significantly slower than the healthy participants (adjusted *M* = 0.487; *SD* = 0.023). Interestingly, the main effect of *Time* was no longer significant [*F*(1, 29) = 0.98; *p* = 0.32; partial *η* ^2^ = 0.033]; however, the *Group × Time* interaction was significant [*F*(1, 29) = 5.51; *p* = 0.026; partial *η* ^2^ = 0.16]; indeed, while the healthy individuals reported higher mean velocity in the post-tool-use (adjusted *M* = 0.508; *SD* = 0.025) condition than the baseline (adjusted *M* = 0.465; *p* = 0.023) [*p* = 0.005], this difference did not emerge in PD patients’ performance [pre-tool use: adjusted *M* = 0.412; *SD* = 0.027; post-tool use: adjusted *M* = 0.399; *SD* = 0.029) [*p* = 0.434]. Moreover, a significant difference emerged between the two groups in the post-tool use (*p* = 0.014) but not in the pre-tool use (*p* = 0.18).

*Peak of velocity*: The main effect of *Group* still remained significant [*F*(1, 29) = 4.92; *p* = 0.034; partial *η* ^2^ = 0.14], since PD patients (adjusted *M* = 0.737; *SD* = 0.043) reported a significant lower peak of velocity than the healthy participants (adjusted *M* = 0.876; *SD* = 0.037). The main effect of *Time* was no longer significant [*F*(1, 29) = 0.046; *p* = 0.83; partial *η* ^2^ = 0.002]; the *Time × Group* interaction was confirmed as not significant [*F*(1, 29) = 0.009; *p* = 0.92; partial *η* ^2^ < 0.001].

*Mean acceleration*: The main effect of *Group* still remained significant [*F*(1, 29) = 13.15; *p* = 0.001; partial *η* ^2^ = 0.31], since PD patients (adjusted *M* = 2.01; *SD* = 0.133) reported a significant lower acceleration than the healthy participants (adjusted *M* = 2.71; *SD* = 0.114). The main effect of *Time* was no longer significant [*F*(1, 29) = 0.008; *p* = 0.93; partial *η* ^2^ < 0.001], and the *Time × Group* interaction was confirmed as not significant [*F*(1, 29) = 0.16; *p* = 0.68; partial *η* ^2^ = 0.006].

*Peak of acceleration*: The main effect of *Group* still remained significant [*F*(1, 29) = 27.95; *p* < 0.001; partial *η* ^2^ = 0.59], since PD patients (adjusted *M* = 5.44; *SD* = 0.36) reported a significant lower peak of acceleration than the healthy participants (adjusted *M* = 8.28; *SD* = 0.31). The main effect of *Time* [*F*(1, 29) = 0.76; *p* = 0.39; partial *η* ^2^ = 0.026] and the *Time × Group* interaction were confirmed again as not significant [*F*(1, 29) = 0.6; *p* = 0.44; partial *η* ^2^ = 0.02].

*Mean deceleration*: The main effect of *Group* still remained significant [*F*(1, 29) = 8.64; *p* = 0.006; partial *η* ^2^ = 0.23], since PD patients (adjusted *M* = −1.11; *SD* = 0.13) reported a significant lower mean of than the healthy participants (adjusted *M* = −1.67; *SD* = 0.11). The main effect of *Time* [*F*(1, 29) = 0.15; *p* = 0.69; partial *η* ^2^ = 0.005] was no longer significant. Interestingly, the *Time × Group* interaction was confirmed as significant [*F*(1, 29) = 7.47; *p* = 0.011; partial *η* ^2^ = 0.2]; indeed, the healthy group reported a significant higher mean deceleration in the post-tool use (adjusted *M* = −1.8; *SD* = 0.12) than the pre-tool use (adjusted *M* = −1.54; *SD* = 0.1) [*p* < 0.001], while no difference emerged in the performance of PD patients (pre-tool use adjusted *M* = −1.113; *SD* = 0.12; post-tool use adjusted *M* = −1.113; *SD* = 0.14) [*p* = 0.88]; moreover, both in pre-tool use (*p* = 0.026) and in the post-tool use (*p* = 0.002), the two groups were significantly different.

*Peak of deceleration*: The main effect of *Group* still remained significant [*F*(1, 29) = 15.83; *p* < 0.001; partial *η* ^2^ = 0.35], since PD patients (adjusted *M* = −3.22; *SD* = 0.21) reported a significant lower peak of deceleration than the healthy participants (adjusted *M* = −4.47; *SD* = 0.18). The main effect of *Time* [*F*(1, 29) = 0.049; *p* = 0.82; partial *η* ^2^ = 0.002] was no longer significant, and the *Group × Time* interaction was confirmed as not significant [*F*(1, 29) = 1.19; *p* = 0.28; partial *η* ^2^ = 0.04].

### The Role of Clinical Characteristics on the Performance in the Pointing Movement Task

Only for the PD group, we studied the relationship between the spatio-temporal parameters and the clinical characteristics of Duration of Disease and UPDRS motor score. The results, reported in Table 2, indicated the absence of any significant relationship, suggesting that the motor performance was no related to the considered clinical characteristics. Moreover, no difference emerged between the PD patients with the left lateralization of symptoms and affected individuals with the right lateralization of symptoms [*p* ≥ 0.081].

**Table 2:** Correlational analyses between the clinical characteristics of Duration of Disease and UPDRS motor score and the spatial-temporal parameters about the performance of PD patients.

			Movement time	Mean velocity	Peak of velocity	Mean acceleration	Peak of acceleration	Mean deceleration	Peak of deceleration
Duration of disease	Pre tool-use	*ρ* *p*	−0.37 0.18	−0.11 0.68	−0.34 0.22	−0.01 0.95	−0.2 0.94	−0.06 0.82	0.05 0.85
	Post tool-use	*ρ* *p*	−0.33 0.24	−0.031 0.91	−0.007 0.98	−0.09 0.75	−0.33 0.23	−0.10 0.72	0.19 0.94
UPDRS motor score	Pre tool-use	*ρ* *p*	0.36 0.2	−0.29 0.3	−0.75 0.79	0.12 0.66	0.02 0.94	−0.41 0.14	−0.132 0.65
	Post tool-use	*ρ* *p*	−0.29 0.3	−0.3 0.28	−0.24 0.4	−0.44 0.88	−0.33 0.24	−0.409 0.14	−0.41 −0.14

*n* = 14.

### Tactile Estimation Task

Neither main effect of *Group* (PD group *M* = 0.58; *SD* = 0.44; controls *M* = 1.38; *SD* = 0.38) [*F*(1, 29) = 1.86; *p* = 0.18; partial *η* ^2^ = 0.06], nor an effect of *Time* (*pre-*tool-use session *M* = 1.12; *SD* = 0.26; *tool-use* session *M* = 0.97; *SD* = 0.31; *post-*tool-use session *M* = 0.87; *SD* = 0.33) [*F*(2, 58) = 1.19; *p* = 0.31; partial *η* ^2^ = 0.039] was found. Moreover, no a significant *Time × Group* interaction [*F*(2,58) = 1.92; *p* = 0.15; partial *η* ^2^ = 0.062] emerged from the analyses. Due to this pattern of result, no further analyses were conducted for controlling the effect of *Age*.

### The Role of Clinical Characteristics on the Performance in the Tactile Estimation Task

Only for the PD group, we studied the relationship between the error reported in the experimental conditions of the Tactile Estimation Task and the clinical characteristics of *Duration of Disease* and *UPDRS motor score*. The results indicated the absence of any significant relationship, suggesting that the tactile estimation judgment was not related to the considered clinical characteristics. Specifically, considering the *Duration of Disease* in years, the relationship was not significant with the error reported in the *pre*-tool-use session [*ρ*(14) = −0.98; *p* = 0.73], in the *tool-use* session [*ρ*(14) = −0.98; *p* = 0.73], and *post*-tool-use session [*ρ*(14) = 0.056; *p* = 0.82]. About *UPDRS motor score*, no significant relationship emerged with the error reported after the *pre*-tool-use session [*ρ*(14) = 0.86; *p* = 0.77], after the *tool-use* session [*ρ*(14) = 0.22; *p* = 0.44], and after the *post-*tool-use session [*ρ*(14) = 0.2; *p* = 0.47]. No difference emerged in the error after the *pre*-tool-use session [*U* = 23; *p* = 0.89], after the *tool-use* session [*U* = 24; *p* = 1], and after the *post*-tool-use session [*U* = 23; *p* = 1] between the PD patients with a left lateralization of symptoms (*pre*-tool-use session *M* = 0.6, *SD* = 0.71; *tool-use*-session *M* = 0.48, *SD* = 0.75; *post*-tool-use session *M* = 0.58, *SD* = 0.77) and those with a right lateralization (*pre*-tool-use session *M* = 0.67, *SD* = 0.49; *tool-use*-session *M* = 0.45, *SD* = 0.52; *post-*tool-use session *M* = 0.73, *SD* = 0.49).

In summary, in all considered spatial-temporal parameters, PD patients were significantly slower than the healthy individuals, as expected (Abbruzzese and Berardelli, 2003); this pattern emerged also when *Age* was taken into account in the analyses. In almost all spatial-temporal parameters (*Movement Time*, *Peak of velocity*, *Mean acceleration*, *Mean deceleration*, and *Peak of deceleration*), a significant difference between the *pre*-tool-use condition (i.e. the baseline) and the *post*-tool-use condition emerged, suggesting an effect of tool-use training on the motor behavior. Interestingly, while the healthy individuals reported higher values of *mean velocity* and higher values of *deceleration* after the *tool-use* training, suggesting then the tool was embodied (Cardinali et al., 2009), such a difference did not emerge in the individuals with PD; this pattern of behavior emerged also when *Age* was taken into account in the analyses, suggesting how the difference in tool embodiment was not explained by age-related effects (Costello et al., 2015; Costello and Bloesch, 2017). On the other hand, tool use did not affect the tactile perceived length of the forearm, as suggested by the results in the Tactile Estimation Task.

## Discussion

In this experimental study, we sought to investigate if a tool can be efficiently embodied in body representation, affecting action, of individuals with diagnosis of PD. According to our results, no changes in spatial-temporal parameters were observed in individuals affected by PD after a tool-use training, mirroring the absence of an effective tool embodiment into body representation. On the contrary, healthy controls had showed changes in velocity components, and specifically in the parameter of deceleration, meaning when individuals are nearest to approach the target after to have achieved the peak of velocity of their movement. This modification might be an effect of a modification in the movement trajectory, as suggested by the changes observed in temporal parameters relative to the amount of time to perform the going movement as well as in the mean velocity parameter. Critically, such a difference did not emerge in the PD patients.

As we have reported in the Introduction, tool embodiment allows investigating the peculiar characteristic of plasticity in body schema (Giummarra et al., 2008; Cardinali et al., 2009; Martel et al., 2016): a tool can be efficiently embodied in body schema, since it is an adaptable and plastic body representation. Multiple pieces of evidence indicated that body schema is altered in different pathological conditions (Berlucchi and Aglioti, 1997; Gallagher, 2001; Haggard and Wolpert, 2005) because of the influence by the aberrant peripheral input, such as in the case of pain (Schwoebel et al., 2001) or hemiplegia (Garbarini et al., 2015). Focusing on PD, it is a disease characterized by a multitude of sensory and motor symptoms, which mostly affect the body and action (Bereczki, 2010; Moustafa et al., 2016). We hypothesize that experiencing motor symptoms (such as tremor, bradykinesia, or rigidity) as well as sensory symptoms (such as pain or numbness of body parts) might alter body schema representation and specifically its plasticity. Indeed, brain processes somatosensory and motor information, which could be altered in PD, to build the complex body representation. As in our knowledge, no previous study had investigated body schema in PD; thus, our hypothesis need to be further explored and supported by future research. For example, it would be very interesting to observe which motor or sensory symptom might have a large impact on body representation. In our sample, we did not find any relationship between motor performance and clinical characteristics measured by UDPRS (Fahn and Elton, 1987), which is the most widely used clinical rating scale for PD in clinical and research setting; moreover, no difference emerged in terms of which body side was most affected by PD symptoms and signs.

Another possible explanation of this result can be traced in the description of body representation: it grounds on the integration of multiple sensory inputs (Gallagher, 2005; Dijkerman and De Haan, 2007). Through the central mechanism of multisensory integration, the different sensory inputs are coordinated together to create a unified and coherent internal representation of the external world (Stein and Meredith, 1993) and of our body (Ehrsson et al., 2012); importantly, the process of multisensory integration (and specifically of visual, tactile, and proprioceptive input) allows also tool embodiment, and specifically that it is part of the body-part-centered representation of space (i.e. peripersonal space), and extended the reachable area (Maravita and Iriki, 2004). Thus, following this hypothesis, the cognitive process of multisensory integration might be intact so that a tool can be efficiently embodied. From an anatomical point of view, basal ganglia play a pivotal role in the multisensory integration process, and specifically of proprioceptive and visual information (Adamovich et al., 2001; Nagy et al., 2006). However, basal ganglia are part of a network primary affected by the degeneration of the dopaminergic neurons of the substantia nigra in PD (Blandini et al., 2000). Indeed, it is not surprising to observe difficulties in the integration of multiple and different sensory inputs in PD (Adamovich et al., 2001; Almeida et al., 2005; Fearon et al., 2015; Ding et al., 2017; Avanzino et al., 2018). For example, Ding et al., 2017 recently hypothesized that the defective integration of proprioceptive-tactile and visual input in PD might impede the emergence of the traditional body illusion of the Rubber Hand in affected individuals. Thus, in the possible difficulties in tool embodiment in PD described in the present work might be due to an alteration of the multisensory integration process, since the anatomical dysfunction at the basal ganglia in PD. Future research needs to explore this topic, adopting more traditional methodological approaches (Calvert and Thesen, 2004; Scarpina et al., 2016) to study multisensory integration in PD. Moreover, the role of PD ideomotor slowness in this capability should be defined (Talsma et al., 2010): indeed, in our study, patients were systematically slower than controls, both in the *pre*- and *post*-tool-use session, which may have masked any change induced by tool use.

Considering the result about the Tactile Estimation Task adopted in the present study to investigate modification in the cognitive representation of arm’s length, no difference emerged between the different experimental sessions. However, this result was observed not only in PD patients, but—against our hypothesis—also in the healthy controls. According to the traditional dualistic model of body representation (Gallagher, 2005; Dijkerman and De Haan, 2007), the Tactile Estimation Task refers to the component of *body image*, that is the perceptual body representation relative to cognition and beliefs, and not specifically involved in action and motor control (Dijkerman and De Haan, 2007). Following this hypothesis, tool use might affect specifically that body representation involved in action (i.e. *body schema*), investigated through the spatial-temporal analyses of *online* movement characteristics, but not the more stable representation of body image (Kammers et al., 2009; Cardinali et al., 2011). However, Cardinali et al., 2009 clearly reported that tool use can modify the perceived length of the arm. In their experiment, participants were asked to point toward different landmarks on the arm to study changes in perceptual body representation after tool-use training. Considering that PD individuals generally show poor accuracy in pointing movements (such as Flash et al., 1992; Adamovich et al., 2001; Pfann et al., 2001), this task might not be completely suitable in this clinical condition. Then, we adopted the Tactile Estimation Task, which allows investigating the body representation through the tactile size perception (Longo, 2015), in the absence of any movements. Nevertheless, both tasks refer to the same mechanism: participants use the representation of their own arm when they estimate the distance between two targets (Tactile Estimation Task) or point towards a target (Cardinali et al., 2009) perceived on the skin surface; thus, in the light of the previous consideration, we would have expected to find a significant difference between the experimental conditions in the Tactile Estimation Task, at least immediately after the tool training condition. However, it could be observed that in our experiment, the participants performed a significantly lower number of movements in all experimental conditions, than the study of Cardinali et al., (2009), perhaps too few to induce a change in the very stable body representation of the body image (Longo and Haggard, 2012). Moreover, it would be noticed that we adopted a very short tool, compared to what was done in previous studies (Serino et al., 2007; Cardinali et al., 2009, 2011; Sposito et al., 2012) in healthy participants; thus, even though our tool was long enough to allow pointing toward a target otherwise unreachable, affecting body schema representation in healthy individuals, it might be too short to change a stable body representation such as the body image (Farnè and Làdavas, 2000; Sposito et al., 2012). Focusing on the nature of the Tactile Estimation Task, it grounds on tactile perception, and specifically on the secondary tactile perception, meaning the process according to which extracting metric information from the skin surface requires additional computational processes over the primary tactile perception (i.e., when the external object presses on the skin) (Dijkerman and De Haan, 2007; Spitoni et al., 2010). However, we would underline that no previous study had measured the tactile threshold (Moseley, 2008) or the secondary tactile discrimination (Spitoni et al., 2010; Scarpina et al., 2014) in PD. Nevertheless, difficulties in sensory discrimination (Sathian et al., 1997; Nolano et al., 2008; Zambito Marsala et al., 2011) have been reported in PD population, requiring future investigation on this topic. Finally, even though it is out of the scope of the present manuscript, we would underline that there are multiple theories about how many body representations are in the brain (De Vignemont, 2007), with consequences on the interpretation of the behavioral data. In the present work, we refer to the traditional dyadic model of *body schema/body image* (Gallagher, 2005; Dijkerman and De Haan, 2007). However, considering the other theoretical frames, we underline that the Tactile Estimation Task might be read as referring to a body structural description (*triadic taxonomy*, e.g., Longo and Haggard, 2010, 2012), and it is a task grounded on that implicit metric body representation that underlies position sense and external tactile localization (Longo, 2015, 2018).

From the preliminary nature of this investigation, some limitations can be recognized. First of all, as previously stated, the number of movements and measurements should be enlarged, even though we need to deal with the negative effect of the well-known non-motor PD symptom of fatigue (Lou et al., 2001; Shulman et al., 2001). Moreover, the task was self-paced; it would be interesting to perform the tasks in different (self-paced vs external-paced) modalities, but it should be taken into account that the overall accuracy and stability of movements can be negatively influenced by attentional processing enhanced by the presence of external cueing in PD (Almeida et al., 2005). Finally, the possible effect related to lateralization of symptoms, meaning which body part side was the most affected by disease, in relation to the dominance handedness, as well as the role of cognitive difficulties in PD (Litvan et al., 2011) and specifically in cognitive estimation (D’Aniello et al., 2015a,b; Scarpina et al., 2017) should be considered. Future research needs to overcome these limitations, where possible.

This study suggests at a novel way to conceive PD sensory motor signs and symptoms: the disease might affect the tool embodiment in cognitive body representation, as a possible secondary effect of altered plasticity of body schema, since the sensory and motor symptoms, or altered multisensory integration process due to the degeneration of dopaminergic neurons in the basal ganglia. Tool embodiment in body representation can extend the potentiality of individual’s action; however, if deficient, it might have remarkable consequences and implications (Giummarra et al., 2008; Mayer et al., 2008; Pazzaglia et al., 2013) on motor behavior, specifically in those clinical conditions like PD, in which the body and action are primarily affected by symptoms.

## Author Contributions

FS conceived the study, collected data, performed the analyses and wrote the main manuscript. NC collected data and performed the kinematic analyses. VC and MG supervised the kinematic analyses. LP recruited patients and performed the neurological examination. AM supervised the recruitment and the neurological examination. All authors reviewed the manuscript.

### Conflict of Interest Statement

The authors declare that the research was conducted in the absence of any commercial or financial relationships that could be construed as a potential conflict of interest.
